# NORA3-WP: A high-resolution offshore wind power dataset for the Baltic, North, Norwegian, and Barents Seas

**DOI:** 10.1038/s41597-022-01451-x

**Published:** 2022-06-24

**Authors:** Ida Marie Solbrekke, Asgeir Sorteberg

**Affiliations:** 1 Geophysical Institute, Bergen Offshore Wind Centre (BOW), University of Bergen, Allègaten 70, 5020 Bergen, Norway; 2 Geophysical Institute, Bjerknes Centre for Climate Research (BCCR), Bergen Offshore Wind Centre (BOW), University of Bergen, Bergen, Norway

**Keywords:** Wind energy, Energy modelling, Energy access

## Abstract

We present a new high resolution wind resource and wind power dataset named NORA3-WP. The dataset covers the North Sea, the Baltic Sea and parts of the Norwegian and Barents Seas. The 3-km Norwegian reanalysis (NORA3) forms the basis for the new dataset. NORA3-WP is an open access dataset intended for use in research, governmental management and for stakeholders to attain relevant wind resource and wind power information in the planning phase of a new wind farm project. The variables are available as monthly data, and provides a climatological overview of 25 wind resource and wind power related variables for three selected turbines for the ocean areas surrounding Norway. In addition, the underlying hourly wind speed data and hourly wind power generation for three selected turbines are also available for higher frequency analysis and case-studies.

## Abstract (summary)

**Table:** 

Measurement(s)	atmospheric wind speed and wind power
Technology Type(s)	Computational technology

## Background & Summary

Offshore wind power continues to take larger portions of the global energy mix. Using good quality data to identify new potential areas for offshore wind power exploitation is important. Offshore wind observations are very sparse and wind power estimations have to rely on stimulated wind speeds. Here we present a high resolution, freely available wind resource and wind power dataset for the offshore areas enclosing Norway called NORA3-WP.

NORA3 forms the basis for the new wind power data set NORA3-WP. NORA3^1^ is most recent high resolution reanalysis from the Norwegian Meteorological institute. NORA3 is generated by a dynamically downscaling of the state-of-the-art reanalysis from the European Center for Medium-Range Weather Forecast (ECMWF) - ERA5^2^. The downscaling is conducted using the high resolution non-hydrostatic numerical weather prediction (NWP) model HARMONIE-AROME^3,4^. NORA3 differs from other existing wind resource datasets in terms of the choice of NWP model used in the downscaling process of ERA5. In contrast to other existing wind energy resource datasets which are created by the Reasearch and Forecasting Model (WRF), NORA3 is created by the NWP model HARMONIE-AROME (Cy 40h1.2). HARMONIE-AROME is a mesoscale-permitting NWP model developed as a European cooperation, and used by many European weather forecast and research institutions^3,4^. The downscaling of ERA5 is performed by solving the fully compressible Euler equations, on a non-staggered horizontal grid in a non-hydrostatic atmospheric mode. NORA3 is extensively evaluated against observations and the host dataset (ERA5)^1,5^. The validation of the wind climatology in NORA3 show that the downscaling of ERA5 resulted in an improved wind resource dataset.

**Fig. 1:**
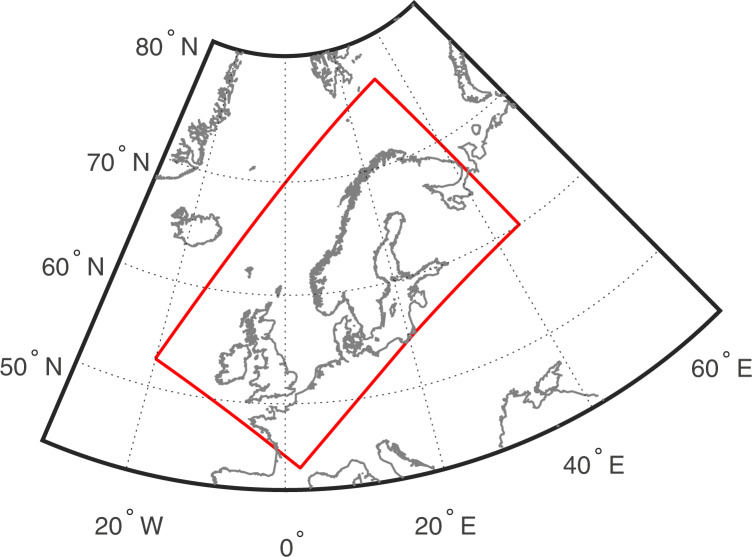
The geographical domain covered by NORA3-WP (red rectangle).

NORA3-WP is generated using hourly wind speeds in several model layers near the surface, together with air temperature and pressure, to estimate relevant wind resource and wind power variables. NORA3-WP consist of statistical measures for 7 wind resource and 18 wind power related variables. The power estimates are generated using three different turbines having different rated powers, turbine diameters, and hub heights (6 MW at 101 m.a.s.l., 10 MW at 119 m.a.s.l., and 15 MW at 150 m.a.s.l.). All variables are stored on a 3 × 3 km horizontal grid covering the North Sea, Norwegian Sea, Baltic Sea and parts of the Barents Sea (See Fig. 1). NORA3-WP spans the period 1996–2019 (will be updated to go back to 1979) and contains monthly values for all variables. In addition, NORA3-WP contains the underlying hourly wind speed and hourly generated wind power data. The hourly variables provides high-frequency data available for more detailed analysis.

NORA3-WP is a state-of-the-art fully open wind resource and wind power dataset facilitated for researchers, decision makers, stakeholders, and investors. The goal of NORA3-WP is to create a dataset for research and for usage in the planning phase of new wind farms. NORA3-WP will give useful information on the climatological features of the wind resource and wind power variables, and provides the underlying hourly wind data for users to perform their own detailed analyses.

NORA3-WP is an open access wind power dataset under the Norwegian Licence for Open Government Data (NLOD). The dataset contributes to the continuously growing ensemble of wind resource datasets (e.g. NEWA^6^, GWA^7^). The growing ensemble of wind resource datasets enables quantification of wind resource uncertainty and we recommend future users to use the NORA3-WP together with other sources of wind resource information.

## Data and Methods

### NORA3: The 3-km Norwegian reanalysis

NORA3 dataset is created by the Norwegian Meteorological Institute by running the non-hydrostatic NWP model HARMONIE-AROME (Cy 40h1.2), solving the fully compressible Euler equations^3,4^. The state-of-the-art reanalysis ERA5 from ECMWF is used as initial and boundary forcing^2^. ERA5 is a global reanalysis product, providing hourly information for 137 vertical layers, and covers the Earth with 0.28125° resolution, corresponding to a horizontal grid of approximately 31 × 31 km. The improved resolution of ERA5 over ECMWF’s former reanalysis ERA-Interim (≈79 km) provides a detailed initial and boundary information in the downscaling process. NORA3 covers large parts of the North Atlantic and the Nordic countries in a 3 × 3 km non-staggered horizontal grid (see Fig. 1 in^5^ for the complete NORA3 domain), dividing the atmosphere into 65 vertical layers. The NORA3 near surface output data are available every hour, and is so far covering the period 1996–2019. When the model integration is finalized (Summer 2022) the NORA3 data will cover the time period from 1979 to present, and will be regularly updated in the coming years. For more details on the generation of the dataset see^1^.

### NORA3-WP: A high-resolution offshore wind power dataset

NORA3-WP is created by estimating wind resource and wind power related variables using NORA3 hourly wind speeds, as well as air temperature and pressure, in several near-surface model levels. The geographical domain covered by NORA3-WP is smaller than the original NORA3-domain. The domain in NORA3-WP covers the eastern parts of the Norwegian sea, the North Sea, the Baltic Sea and part of the Barents Sea (see Fig. 1 for the NORA3-WP domain). The horizontal grid resolution is 3 × 3 km. The dataset have 652 grid points in the x-direction (longitude) and 1149 grid points in y-direction (latitude). Wind power variables are calculated for three different turbines having different turbine specifications (see section on wind turbine specifications) and the wind resource variables are available at the same heights: 101 *m.a.s.l*., 119 *m.a.s.l*. and 150 *m.a.s.l*. The NORA3-WP data covers the period from 1996 to 2019, and the variables are stored as monthly data. To facilitate more detailed analysis with increased temporal resolution, hourly wind speed and hourly generated wind power for the different turbines are also available.

### Wind turbine specifications

**Fig. 2:**
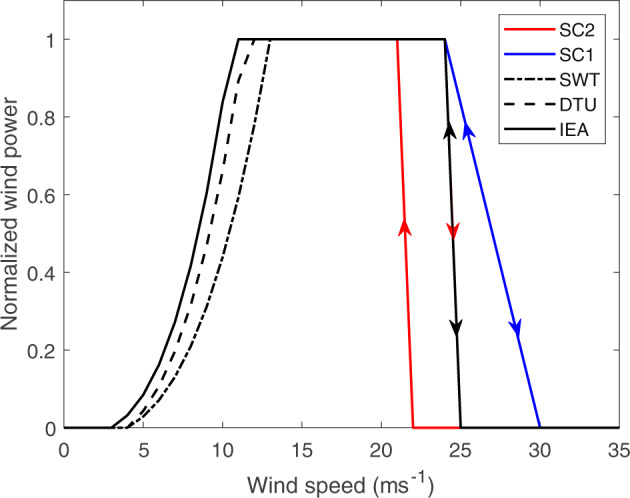
Power curves for the three turbines (SWT-6.0–154, DTU-10.0-RWT, IEA-15–240-RWT). In addition, the figure illustrates how the high wind speed end of the power curve changes when storm control 1 (SC1) and storm control 2 (SC2) are included. The arrows indicate how the different power curves shut down (arrow down) and restart (arrow up) at high wind speeds.

Specifications about the three turbines used to create the wind power related variables of NORA3-WP are listed in Table 1. SWT-6.0–154 from Siemens is the floating three bladed electricity generator used in Hywind Scotland, the first floating wind farm in the world^8^. SWT-6.0–154 has a rated power of 6 MW with a rotor diameter and hub-height of 154 m and 101 m.a.s.l., respectively. DTU-10.0-RWT is the widely used reference wind turbine from the Technical University of Denmark (DTU)^9^. The rated power of DTU-10.0-RWT is 10 MW and the rotor diameter and hub-height corresponds to 178.3 m and 119 m.a.s.l., respectively. We have also used a new offshore reference turbine from the National Renewable Energy Laboratory, IEA-15–240-RWT^10^. This large turbine with a rotor diameter of 240 m produces 15 MW at rated power at the hub-height of 150 m.a.s.l. See Fig. 2 for the three normalized power curves considered in this study. The advantage of the reference turbines is the open access to all design parameters. The easy access to key design parameters makes it easier to explore the technical specifications, and enables and facilitates collaboration between the industry and the research community.

**Table 1:** Turbine specifications for the three turbines used to generate the wind power related variables in NORA3-WP.

	SWT-6.0-154	DTU-10.0-RWT	IEA-15-240-RWT
Rated power, *C*_*r*_ (*W*)	6 000 000	10 000 000	15 000 000
Hub height (*m*)	101	119	150
Rotor diameter (*m*)	154	178.3	240
Specific rated power *C*_*r*_*/A* (*Wm*^−2^)	161.1	200.3	165.8
cut-in (*ms*^−1^)	4.0	4.0	3.0
rated (*ms*^−1^)	13.0	11.4	10.59
cut-out (*ms*^−1^)	25.0	25.0	25.0

When calculating the wind power related variables we assume a stand-alone wind turbine experiencing no wind farm effects or other disturbances that can reduce the power production.

### Statistics

This section contains the statistics used in the generation of NORA3-WP, here expressed by x (x can be e.g. wind speed, power density, wind power etc,.):

#### Arithmetic mean ($\bar{{\boldsymbol{x}}}$) of x:

1 $$\bar{x}=\frac{1}{n}\mathop{\sum }\limits_{t=1}^{n}x(t)$$

#### Maximum value (x_*max*_) of x:

2 $${x}_{max}=max(x)$$

#### Percentiles (x_*yy*_) of x

After sorting x in ascending order, yy-percentile of x (*x*_*yy*_) gives us the value of x where yy percent of the data falls at and below *x*_*yy*_, and (1-yy) percent of the data falls above *x*_*yy*_.

#### Weibull distribution, scale (a), and shape (b) parameters of x

The probability density function *f*(*x; a, b*) for a Weibull variable x, with scale and shape parameters *a* and *b*, respectively, is:

3 $$f(x;a,b)=\left(\begin{array}{ll}\frac{b}{a}\left({\frac{x}{a}}^{(b-1)}{e}^{{(x/a)}^{b}}\right), & x\ge 0\\ 0, & x < 0\end{array}\right.$$

By fitting a Weibull probability distribution function to the data in x we are able to get the maximum likelihood estimates of the Weibull scale ($\widehat{a}$) and shape ($\widehat{b}$) parameters:

4 $$\widehat{a}={\left[\frac{1}{n}\mathop{\sum }\limits_{t=1}^{n}x{(t)}^{\widehat{b}}\right]}^{\frac{1}{\widehat{b}}},$$

5 $$\widehat{b}=\frac{n}{\frac{1}{\widehat{a}}{\sum }_{t=1}^{n}\,x{(t)}^{\widehat{b}}log(x(t))-{\sum }_{t=1}^{n}\,log(x(t))},$$

n is the number of samples (here: hours). The scale parameter (*a*) gives the height of the distribution. A relative large *a* corresponds to a high and narrow Weibull distribution. The shape parameter (*b*) gives the shape of the distribution. If *b* < 3 the data distribution is right-skewed, with a long tail to the right of the mean.

#### Weibull standard deviation (σ_***x***_) of x:

6 $${\sigma }_{x}=a\sqrt{\Gamma \left(\frac{2}{b}+1\right)-\Gamma \left(\frac{1}{b}+1\right)}$$

where $\Gamma (n)={\int }_{0}^{\infty }{e}^{-x}{x}^{n-1}dx$ is the Gamma function evaluated at $\left(\frac{2}{b}+1\right)$ and $\left(\frac{1}{b}+1\right)$.

## Data Records

This section contains explanations and calculations for each data record associated with NORA3-WP^11^. Each subsection describes a variable in the dataset. The heading of the subsection corresponds to the variable names in Tables 2 and 3.

**Table 2:** The wind speed related variables available in NORA3-WP.

Variable	Stat	unit	X grid × Y grid × time	height (m)
Hourly wind speed	—	*ms*^−1^	652 × 1149 × *h*_*month*_	hh 1, hh 2, hh 3
Wind speed	Mean, 25-, 50-, 75-, 95-percentile, std, max	*ms*^−1^	652 × 1149 × *n*_*month*_	hh 1, hh 2, hh 3
Exponential power law coefficient (*α*)	Mean	—	652 × 1149 × *n*_*month*_	10–100, 50–100, 100–250
Weibull wind speed parameters	Scale, shape	*ms*^−1^,-	652 × 1149 × *n*_*month*_	hh 1, hh 2, hh 3
Prevailing wind direction sector	Mean	degrees	652 × 1149 × *n*_*month*_	100
Vertical wind shear	Mean, max	*ms*^−1^	652 × 1149 × *n*_*month*_	50–100, 100–250
Wind speed absolute ramp-rate (ARR)	Mean, max	*ms*^−1^	652 × 1149 × *n*_*month*_	hh 1, hh 2, hh 3

All the listed variables are available as monthly means of hourly data for each months in the study period, except “Hourly wind speed” which is available as hourly output for each month. *h*_*month*_ is the number of hours in the current month, *n*_*month*_ is the total number of months in the study period (288 months), hh1, hh2, and hh3 corresponds to the hub height of the three turbines, 101 m.a.s.l., 119 m.a.s.l., and 150 m.a.s.l., respectively (see Table 1).

**Table 3:** Wind power variables available for download in NORA3-WP.

Variable	Stat	unit	X grid × Y grid × time	height (m)
Power density, *P*_*d*_	Mean	*Wm*^−2^	652 × 1149 × *n*_*month*_	hh 1, hh 2, hh 3
Power capture, *P*_*c*_	Mean	*Warea*^−1^	652 × 1149 × *n*_*month*_	hh 1, hh 2, hh 3
Hourly generated power, *P*_*w*_	—	W	652 × 1149 × *h*_*month*_	hh 1, hh 2, hh 3
Power generated, *P*_*w*_	Mean, 25−, 50−, 75-percentile	W	652 × 1149 × *n*_*month*_	hh 1, hh 2, hh 3
Power generated, density correction	Mean	W	652 × 1149 × *n*_*month*_	hh 1, hh 2, hh 3
Power generated, SC1 *P*_*w,SC*1_	Mean	W	652 × 1149 × *n*_*month*_	hh 1, hh 2, hh 3
Power generated, SC2 *P*_*w,SC*2_	Mean	W	652 × 1149 × *n*_*month*_	hh 1, hh 2, hh 3
Power capture coefficient, *P*_*cc*_	Mean	%	652 × 1149 × *n*_*month*_	hh 1, hh 2, hh 3
Generated power absolute ramp-rate (ARR)	Mean, max	W	652 × 1149 × *n*_*month*_	hh 1, hh 2, hh 3
Time fraction cubed power (${u}_{ci}\le u < {u}_{r}$)	—	%	652 × 1149 × *n*_*month*_	hh 1, hh 2, hh 3
Time fraction rated power (${u}_{r}\le u < {u}_{co}$)	—	%	652 × 1149 × *n*_*month*_	hh 1, hh 2, hh 3
Time fraction zero power ($u < {u}_{ci},u\ge {u}_{co}$)	—	%	652 × 1149 × *n*_*month*_	hh 1, hh 2, hh 3
Time fraction zero power, SC1 ($u < {u}_{ci},u\ge {u}_{co}$)	—	%	652 × 1149 × *n*_*month*_	hh 1, hh 2, hh 3
Time fraction zero power, SC2 ($u < {u}_{ci},u\ge {u}_{co}$)	—	%	652 × 1149 × *n*_*month*_	hh 1, hh 2, hh 3
Capacity factor	—	%	652 × 1149 × *n*_*month*_	hh 1, hh 2, hh 3
Full load hours	—	h	652 × 1149 × *n*_*month*_	hh 1, hh 2, hh 3
Full load hours, SC1	—	h	652 × 1149 × *n*_*month*_	hh 1, hh 2, hh 3
Full load hours, SC2	—	h	652 × 1149 × *n*_*month*_	hh 1, hh 2, hh 3

All the listed variables are monthly data, except “Hourly generated power” which is hourly data. “SC1” is the storm control 1, “SC2” is the storm control 2 scenario with high wind hysteresis. *h*_*month*_ is the number of hours in the current month, *n*_*month*_ is the total number of months in the study period (288 months), “hh1”, “hh2”, and “hh3” correspond to the hub height of the three turbines, 101 m.a.s.l., 119 m.a.s.l., and 150 m.a.s.l., respectively (see Table 1).

### Wind speed variables

The wind speed related variables included in the NORA3-WP dataset are listed in Table 2 and described below.

#### Wind speed

Both hourly, monthly mean, monthly maximum and monthly percentiles (25-, 50-, 75-, and 95-percentile) of the wind speed are available. For the wind speed to be valid at the relevant turbine hub heights the NORA3 wind speed data at height *z*_1_ (*z*_1_ = 50*m.a.s.l*., *z*_1_ = 100*m.a.s.l*. and *z*_1_ = 250*m.a.s.l*.) are interpolated to the turbine hub height (*z*_2_) using the exponential relation with hourly varying power law coefficient *α*(*t*):

7 $${u}_{{z}_{2}}(t)={u}_{{z}_{1}}(t){\left(\frac{{z}_{2}}{{z}_{1}}\right)}^{\alpha (t)},$$

Equations 1 and 2 are applied to Eq. 7 to obtain monthly mean and maximum wind speed values, respectively.

#### Exponential power law coefficient, *α*

The exponential power law exponent (*α*) modifies the wind speed profile and is a function of the undisturbed wind speed, atmospheric stability, and surface roughness. The hourly power law exponent is obtained by solving Eq. 7 for *α*;

8 $$\alpha (t)=\frac{ln(\frac{{u}_{{z}_{2}}(t)}{{u}_{{z}_{1}}(t)})}{ln(\frac{{z}_{2}}{{z}_{1}})}$$

where *Z*_1_ and *Z*_2_ correspond to the two layers within the wind shear is calculated. *α* is calculated between 10 and 100 *m.a.s.l*, 50 and 100 *m.a.s.l*, and 100 and 250 *m.a.s.l* depending on the hub height of the wind turbine. Equation 1 is applied to Eq. 8 to obtain the monthly mean values of the power law exponent.

#### Weibull wind speed parameters

The wind speed distribution can be approximated through the Weibull **scale factor (*a*)** and **shape factor (*b*)** parameters^12,13^. Using these factors an approximated wind distribution for a each month can be generated without having to download the hourly data. The combination of *a* and *b* brings information about the fraction of the data that falls between cut-in and cut-out wind speed. Together, *a* and *b* are indicative of the wind power production without using a specific wind turbine. Nevertheless, we want to stress that using the hourly wind speed data for wind power production estimates will give a more realistic power output compared to a Weibull distribution fit. Monthly scale and shape factors are calculated for each grid cell by using Eq. 4 and Eq. 5. Typical values for the average scale and shape parameters at the Norwegian offshore area are 9ms^−1^ ≤ *a* ≤ 12ms^−1^ and 1.7 ≤ *b* ≤ 2.7.

#### Vertical wind shear

The vertical distribution of the wind speed with height is relevant in wind energy application. Wind turbine height and rotor diameter are parameters that have been continuously increasing. As a consequence, the wind turbine rotor sweeps a large portion of the atmospheric boundary layer where the wind changes rapidly with height. In NORA3-WP the atmospheric vertical wind shear (${u}_{{z}_{2}}-{u}_{{z}_{1}}$) are calculated between the most relevant model layers for wind energy; between 50 and 100 m; and between 100 m and 250 m. The vertical wind shear (*δu*) is how much the wind speed changes over a given height interval:

9 $$\delta u={u}_{z2}-{u}_{z1},$$

where *z*_1_ and *z*_2_ correspond to the two layers within which the wind shear is calculated. Equation 1 and Eq. 2 are applied to the wind shear (${u}_{{z}_{2}}-{u}_{{z}_{1}}$) to obtain the monthly mean and maximum values, respectively.

#### Prevailing wind-direction sector

Mapping the wind direction climatology is important for wind farm layout. Wind turbine technology allow the wind turbines to yaw to face the main wind direction. However, the wind farm layout are optimized according to the wind direction climatology. The wind direction is calculated from the original NORA3 data taken into account that the NORA3 data is using a rotated grid configuration. NORA3-WP contains the monthly mean wind direction for the prevailing wind-direction sector. This is done by first finding which of the eight 45-degree wind sectors (the sector splitting starts at 0 degrees) is the most frequent in terms of hourly directions. Monthly averaging (Eq. 1) is then applied to all the winds direction events contained in the prevailing sector. Directions are given as where the wind blows from.

#### Wind speed absolute ramp-rate (ARR)

Wind speed variability is one of the major challenges related to wind power generation. The wind speed variability combined with the power curve generates an even more intermittent wind power production. This fluctuating wind power output requires a highly flexible power system^14^. How much the wind speed changes from one hour to the next is a good measure of the variability in the wind speed, and is here given by the hourly absolute ramp rate (ARR(t)).

10 $$AR{R}_{u}(t)=| u(t)-u(t+1)| $$

Taking the mean (*M*_*ean*_ *ARR*) and maximum (*M*_*ax*_ *ARR*) values of the ARR by using Eq. 1 and Eq. 2, respectively, will quantify the hourly absolute temporal wind speed variability at hub height for each month.

### Wind power variables

The wind power related variables in NORA3-WP are listed in Table 3, and described below.

#### Power density

The total power in the atmosphere for a wind turbine to extract is the power density at hour t (*P*_*d*_ (*t*)), and is expressed by the following relation:

11 $${P}_{d}(t)=\frac{1}{2}\rho (t)u{(t)}^{3},$$

which gives us the amount of kinetic energy contained in the air per square meter (*Wm*^−2^), and is a function of the air density (*ρ*) and wind velocity (*u*) at hub height. The hourly air density at the three hub heights is calculated using the hourly temperature and pressure by assuming hydrostatic balance in the following way:

12 $$\rho {(t)}_{hub}={\rho }_{s}{e}^{\left(\frac{-gz}{{R}_{d}{T}_{avg}}\right)},$$

where ${\rho }_{s}=\frac{{p}_{s}}{{R}_{d}{T}_{2m}}$ is the density at the surface, *R*_*d*_ is the gas constant for dry air, *T*_2*m*_ is the temperature at 2 m, *p*_*s*_ is the surface pressure, ${T}_{avg}=\frac{1}{2}\left({T}_{2m}+{T}_{z}\right)$ is the bulk-average between the temperature at 2 m and the temperature at the hub height. ${T}_{z}={T}_{2m}\frac{dT}{dz}\left(z-2\right)$ is the temperature at height z. $\frac{dT}{dz}=-6.5{\rm{K}}/{\rm{km}}$.

#### Power capture

Equation 11 gives the total kinetic energy in the air per square meter. Since the extracted wind energy is a function of the turbine diameter, and hence the rotor disk area (A), we multiply Eq. 11 by the sweep area A to get the theoretical power (W) captured by the rotor disk at hour t:

13 $${P}_{c}(t)=\frac{1}{2}\rho (t)u{(t)}^{3}A.$$

The monthly mean of *P*_*c*_ is calculated using *P*_*c*_ in Eq. 1.

#### Power generated

Turbine specifications pose limitations to the theoretical power potential. The generated power at hour t (*P*_*w*_ (*t*)) is the wind power that can be produced for a specific turbine, and is given by the installed (rated) capacity (*C*_*r*_) multiplied by the normalized non-linear power conversion function (*P*_*w,n*_ (*t*));

14 $${P}_{w}(t)={C}_{r}{P}_{w,n}(t),\quad {P}_{w,n}(t)=\left(\begin{array}{ll}0, & u(t) < {u}_{ci}\\ \frac{u{(t)}^{3}-{u}_{ci}^{3}}{{u}_{r}^{3}-{u}_{ci}^{3}}, & {u}_{ci}\le u(t) < {u}_{r},\\ 1, & {u}_{r}\le u(t) < {u}_{co}\\ 0, & {u}_{co}\le u(t).\end{array}\right.$$

where *u*(*t*) is the wind speed at hour t, *u*_*ci*_ is the cut-in wind speed, *u*_*r*_ is the rated wind speed, and *u*_*co*_ is the cut-out wind speed. The specification of these numbers varies for the different turbines and can be seen in Table 1. See also Fig. 2 for the different power generation curves.

Equation 1 is applied to Eq. 14 to derive the monthly mean wind power production. In addition, the 25-, 50-, and 75-percentile are calculated to obtain the typical monthly wind power output, and monthly range of wind power production in each grid cell. In addition to the power estimation described above, the dataset contains three different ways of estimating the generated power. We supply NORA3-WP with these additional power generation methods to consider density corrections and storm control options. They are described below.

#### Power generated, density correction

The atmospheric wind power is directly proportional to the air density (see Eq. 11). For inter-comparison of power production curves from different wind turbines and for expressing the power production as a function of the wind speed only, the power curves are calculated using reference air density (*ρ*_*ref*_). At *T* = 15° C and *P*_0_ = 1013.25 hPa the reference atmospheric density is $\rho =1.225\frac{kg}{{m}^{3}}$. However, the atmospheric density is not constant. Whenever the air density deviates from the reference air density will result in erroneous power production estimates, on average 1–2%. To include density variations (both temporal, spatial and changes with height) while retaining the single-variable dependency of the power curve, we correct the wind speed at hub height (*u*_*cor r*_). The corrected wind speed is here expressed as a function of the reference air density (*ρ*_*ref*_) and the site-specific air density at hub height (${\rho }_{{z}_{2}}$). The density corrections follow the work by Svenning (2010)^15^:

15 $${u}_{corr}(t)=\left(\begin{array}{ll}{u}_{{z}_{2}}(t){\left(\frac{{\rho }_{ref}}{{\rho }_{{z}_{2}}(t)}\right)}^{\frac{1}{3}} & {u}_{{z}_{2}}(t)\le 8m{s}^{-1},\\ {u}_{{z}_{2}}(t){\left(\frac{{\rho }_{ref}}{{\rho }_{{z}_{2}}(t)}\right)}^{\frac{1}{3}\left(1+\frac{{u}_{{z}_{2}}(t)-8}{5}\right)} & 8m{s}^{-1} < {u}_{{z}_{2}}(t) < 13m{s}^{-1},\\ {u}_{{z}_{2}}(t){\left(\frac{{\rho }_{ref}}{{\rho }_{{z}_{2}}(t)}\right)}^{\frac{2}{3}} & {u}_{{z}_{2}}(t)\ge 13m{s}^{-1},\end{array}\right.$$

where *u*_*cor r*_ and ${u}_{{z}_{2}}$ are the density-corrected wind speed and site-specific wind speed at hub-height (*z*_2_), respectively. However, this density correction of the power curve will result in a disturbed relationship between the power production and the wind speed. If ${\rho }_{z2} < {\rho }_{ref}$ the power production as a function of *u*_*cor r*_ will be underestimated, and vice versa if ${\rho }_{z2} > {\rho }_{ref}$. Therefore, for the density corrected wind power to be valid at the original and unaffected wind speed, the wind power is linearly interpolated back to the original wind speeds (see^15^ for details).

#### Storm control of generated wind power

A storm control is typically implemented in the control software of a wind turbine to increase the stability of the power output for wind events close to the cut-out limit. The mean wind speed at the Norwegian offshore areas range between 9–11 ms^−1^. A high mean wind speed increases the risk of wind events where the wind speed exceeds the cut-out limit. However, for each grid point in NORA3-WP the number of zero-events caused by too high wind will vary. More than 22% of the offshore grid points experience these unwanted zero-events 1–2% of the time.

The ability to produce power during wind speed events exceeding the cut-out limit is important for the production credit and will increase the profitability of the wind farm. In addition, the wind power variability due to start-up and shut-down of the wind power production at high wind speeds requires a highly flexible power system^14^. Here, we introduce two methods to cope with the aforementioned challenges related to power production at high wind speeds.

##### Power generated using smooth shut-down and restart at high wind speeds, SC1

Storm control 1 (SC1) is a turbine control strategy which increases the generated wind power at high wind speed and reduces power intermittency. Instead of an abrupt shut-down of the power production when the wind speed exceeds the cut-out limit a smooth shut-down and start-up procedure is practiced. The power production implementing the SC1 method follows the power conversion equation (Eq. 14) until $u(t)\ge {u}_{co}$, then the power production follows a linear decrease until a new and higher cut-out limit (*u*_*co,new*_, here: 30 ms^−1^) is reached (see blue line in Fig. 2). In this study, when ${u}_{co}\le u(t) < {u}_{co,new}$, SC1 is calculated as follows:

16 $${P}_{w,s}(t)=\frac{{u}_{co,new}-u(t)}{{u}_{co,new}-{u}_{co}},\quad \;{u}_{co}\le u(t) < {u}_{co,new}$$

where *u*(*t*) is the wind speed at hour t, *u*_*co*_ is the cut-out limit for the turbine in question, and *u*_*co,new*_ is the new and higher cut-out limit.

##### Power generated using high wind hysteresis, SC2

Power generation using high wind hysteresis is here called Storm control 2 (SC2). SC2 is a solution used to avoid frequent wind power shut-downs and start-ups when the wind speed fluctuates around the cut-out limit. When the wind speed exceeds the cut-out limit, the SC2 involves a termination of wind power generation until the wind speed is below a given wind speed threshold (*u*_*P,start*_), lower than the cut-out limit:

17 $${u}_{p,start}={u}_{co}-{u}_{incr}$$

*u*_*incr*_ is a wind speed increment in the order of $\frac{{u}_{co}}{10}$ (here: 3 *ms*^−1^). For $u(i) < {u}_{co}$ the wind power generation follows Eq. 14. See Fig. 2 for the power generation curve using SC2.

#### Power capture coefficient

How much of the available power embedded in the air per area that actually generates wind power is here called the power-capture coefficient *P*_*cc*_. This coefficient describes how efficient a specific turbine is at extracting the available power in the air. *P*_*cc*_ is a non-dimensional number that gives the fraction of produced power (*P*_*w*_) per available power (*P*_*c*_):

18 $${P}_{cc}=\frac{{P}_{w}}{{P}_{c}}$$

The monthly mean of *P*_*cc*_ is calculating using Eq. 1. In addition, the monthly maximum *P*_*cc*_ is calculating using Eq. 2.

#### Generated power absolute ramp-rate (ARR)

The hourly wind power ramp-rate measures how much the wind power generation changes from one hour to the next ($AR{R}_{{P}_{w}}(t)$). It is calculated in the same way as the wind speed ramp rate using the generated power (section 3) instead of the wind speed.

#### Capacity factor

The capacity factor (*C*_*f*_) is a common performance measure of a wind turbine or a wind farm. *C*_*f*_ is defined as the average power production divided by the rated power production (*C*_*t*_):

19 $${C}_{f}=\frac{\frac{1}{n}{\sum }_{t=1}^{n}{P}_{w}(t)}{{C}_{t}}$$

where *C*_*f*_ is the monthly capacity factor, and n is the number of hours in a month.

#### Full load hours

Full load hours (FLH) is another performance measure. FLH is calculated by taking the monthly sum of the power production divided by the rated power production (*C*_*r*_). The resulting quantity provides the number of hours the turbine has to operate at rated capacity to produce the monthly power production delivered by the specific wind turbine:

20 $$FLH=\frac{{\sum }_{t=1}^{n}{P}_{w}(t)}{{C}_{r}}$$

where FLH is the monthly full load hours, hence n is the numbers of hours in a month. Full load hours using the two storm control methods are also calculated (**Full load hours, SC1** and **Full load hours, SC2**).

#### Power production categorization

It is of major importance for the wind power profitability to quantify the time fraction the power production falls into the following four categories:

- **Time fraction zero power low wind** - time fraction when the wind power is zero due to wind speeds below the cut-in wind speed limit: $u < {u}_{ci}$
- **Time fraction cubed power** - time fraction when the power production is proportional to the wind speed cubed: ${u}_{ci}\le u < {u}_{r}$
- **Time fraction rated power** - time fraction of constant rated wind power production: ${u}_{r}\le u < {u}_{co}$)
- **Time fraction zero power high wind** - time fraction of terminated wind power due to wind speeds exceeding the cut-out limit: ${u}_{co}\le u$

The categorical quantification is done by counting hours where power production falls into each of the four categories (*h*_*cat*_) and normalizing the sum of hours by the total numbers of hours in a month (*h*_*tot*_) according to the equation below:

21 $${f}_{{P}_{w},cat}=\frac{{h}_{{P}_{w},cat}}{{h}_{{P}_{w},tot}}100{\rm{ \% }}$$

In addition to the fractional time the production falls into the four categories above, the total amount of zero power production for the scenario of a smooth shutdown (**Time fraction zero generated power, SC1**) and high-wind hysteresis (**Time fraction zero generated power, SC2**) are also quantified according to Eq. 21:

## Technical Validation

The NORA3 near surface wind estimates are extensively validated against observations and compared against the ERA5 reanalysis by Haakenstad *et al*.^1^. For offshore observations the NORA3 wind estimates were shown to be better than the wind estimates from ERA5 for all months and for all investigated percentiles of wind speed. Monthly wind speed biases were typically reduced from 6–8% to 3–5%. The improvement was particularly pronounced for strong winds, where the bias was reduced from 10–20% to 2–4%, while the bias reduction for median winds typically was reduced from 7–8% to 3–4%. In addition, improvements in coastal winds influenced by topography were shown to be significantly larger than for the offshore stations. Thus, the downscaling of ERA5 resulted in an improved wind resource dataset.

**Fig. 3:**
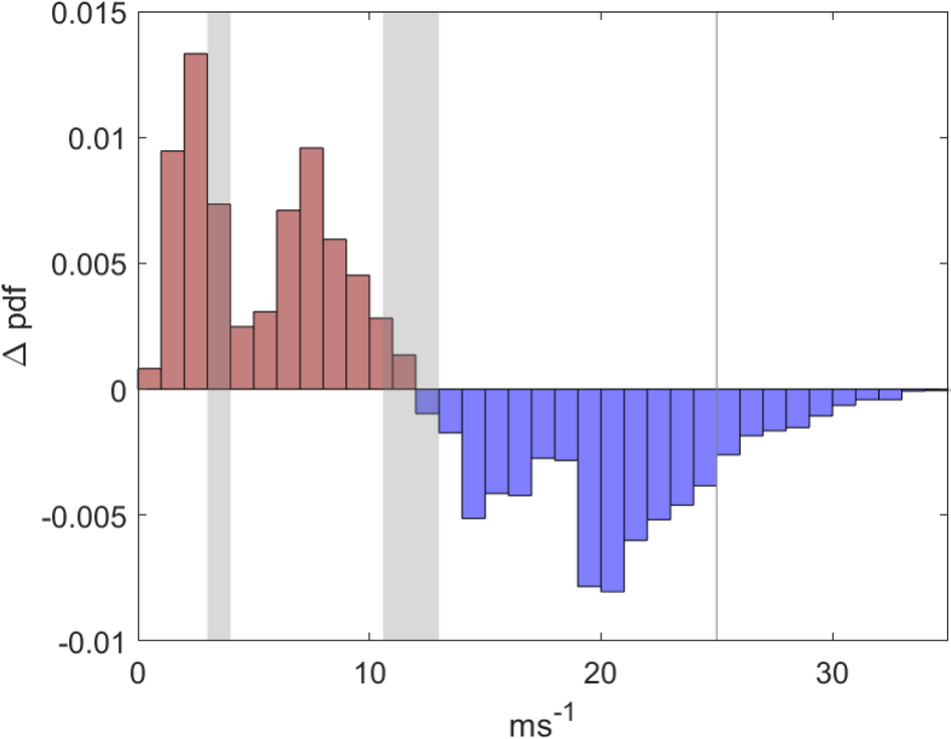
Differences between NORA3 and observational wind speed probability density functions (Δ*pdf* = *pdf*_*mod*_ − *pdf*_*obs*_) for Sleipner (an offshore oil- and gas platform in the North Sea. Lat: 58.36; lon: 01.91). When Δ*pdf* = 0.01 the probability that the given wind speed will occur is 1% higher in the model output. Red (blue) colored bars correspond to Δ*pdf* = *pdf*_*mod*_ − *pdf*_*obs*_ > (<)0. The gray area in the middle corresponds to the range of rated wind speeds for the three turbines used in this study (SWT-6.0-154, DTU-10.0-RWT, IEA-15-240-RWT). The gray area to the left (right) is the range of the cut-in (cut-out) wind speed limits for the three turbines.

The NORA3 dataset was not created specifically for wind power purposes. An in-depth validation of the usefulness of the NORA3 wind dataset in estimating wind power related variables was conducted in detail in Solbrekke *et al*.^5^. One of the main findings in^5^ is that NORA3 wind speeds are typically 5% (0.5 *ms*^−1^) lower than observed wind speeds. The simulated winds are somewhat biased towards an excess of low wind speed events (u < 11–13 ms^−1^) and biased towards too few high wind speed events (u>11–13 ms^−1^) (see Fig. 3). Wind speeds in the order of 11–13 *ms*^−1^ is the wind speed interval where offshore wind turbines generally starts the rated power production. The overestimation of the low wind speed events and the underestimation of the high wind speed events lead to an underestimation of offshore wind power of 10–20% (equivalent to an underestimation of 3 percentage points in the capacity factor).

The validation in^5^ also reveal a slightly lower hourly variability in the NORA3 winds compared to the observational data. Also, there are too few occurrences of unwanted zero-events (zero wind power production due to either too low or too high wind speeds) in the model, and the corresponding event-duration are too long. Hence, the total risk of having an unwanted zero-event is slightly overestimated by NORA3.^5^ concluded that the NORA3 data was well suited for wind power estimates but gives slightly conservative estimates of the offshore wind resources compared to observational-based estimates. The model limitations and weaknesses should be kept in mind when using the dataset as an offshore wind power planning tool.

## Usage Notes

The original NORA3 dataset can be downloaded here: https://thredds.met.no/thredds/projects/nora3.html The NORA3-WP^11^ dataset can be accessed here: https://archive.sigma2.no/pages/public/searchResult.jsf.

The NORA3-WP dataset are available as netCDF4-files (.nc-files). The wind resource parameters available for download are listed in Table 2. The wind power related variables can be found in Table 3.

NORA3-WP is structured and stored following the naming convention of Tables 2 and 3. Each file contains monthly data for all the available years (1996–2019). The hourly data are stored as yearly files due to the file size.

The user should note that onshore data are also available in the files. However, validation of NORA3 over land towards wind power usage is not conducted yet and the choice of turbines used in the wind power estimations is not relevant for land-based sites. To exclude the land grid points from the dataset the land-area-fraction matrix can be used (excl_land_NORA3WP.nc).

### Example of usage

This section provides two examples of usage for the NORA3-WP dataset. The NORA3-WP dataset can be used for different purposes by wind power stakeholders, decision makers, politicians, researchers, journalists etc. Data for a specific variable can be viewed in spatial maps for a smaller region or the whole NORA3-WP domain. Plotting the data in maps gives a spatial overview of the variable considered. The maps provide information on areas suitable for wind energy exploitation. As an alternative to spatial maps, the data can be used to provide temporal information for one or several variables for a specific site, which might be useful to follow the time evolution of the variable in question.

#### Climatology

**Fig. 4:**
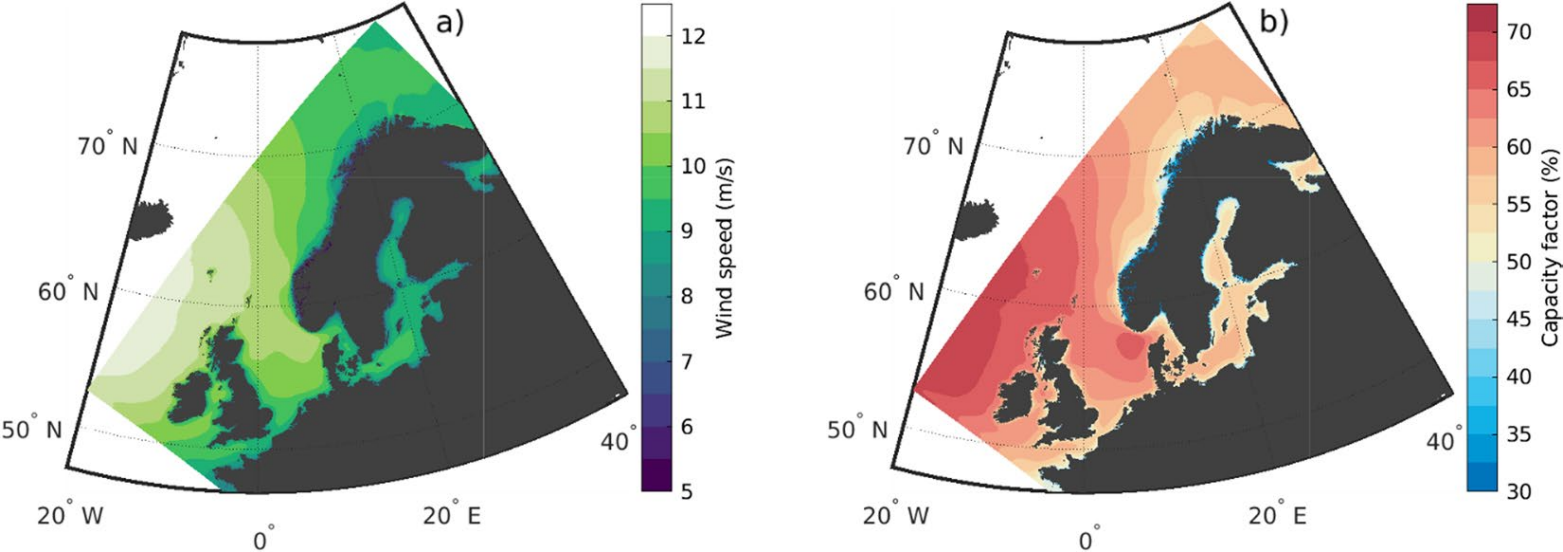
Climatology for the years 1996–2019 of wind speed at 150 m.a.s.l. (**a**) and the corresponding capacity factor for the IEA-15–240-RWT reference turbine (**b**).

An important usage of NORA3-WP is climatological insight into potential wind power production. Figure 4 illustrates the climatology of the wind speed at 150 m.a.s.l. for the years 1996–2019 (a), and the corresponding wind power capacity factor (CF) for the IEA-15–240-RWT turbine (b). Panel a) demonstrates that the mean wind speeds in the area are very high; between 10–12 ms^−1^ in the southern and western regions and slightly lower in the northern and eastern parts (8–10ms^−1^). The climatological CF (panel b) shows similar spatial patterns with highest values in the southern and western areas, with typically 10–15 percentage point lower CF in the northern and eastern regions. Note that the areas with highest wind speeds not necessarily coincide with the areas of the highest capacity factor.

#### Case studies

**Fig. 5:**
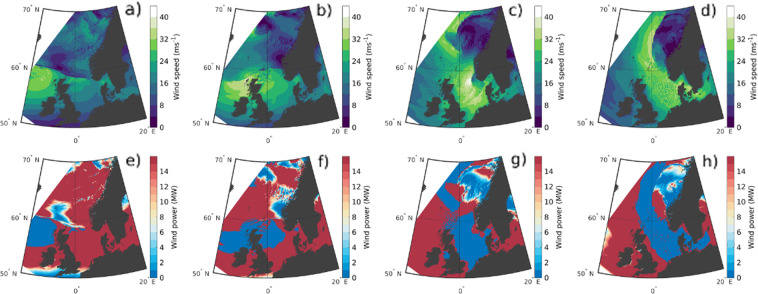
Time evolution of the wind speed (**a**–**d**) and generated wind power (**e**–**h**) for the 5th of December 2013 when a strong low-pressure system struck the North Sea. (**a**) and (**e**) correspond to the 5th of December 2013 at 00UTC; (**b**) and **f**) the 5th at 06UTC; (**c**) and (**g**) the 5th at 12UTC; and (**d**) and (**h**) the 5th at 18UTC.

Another application for the NORA3-WP dataset are case-studies with limited spatial and temporal duration. One specific incident is the storm surge that struck the North Sea on the 5th of December 2013. Figure 5 shows the time evolution of the wind speed and the corresponding wind power production during the 5th of December 2013. In panel a) at 5th of December at 00UTC the wind power production is rated as the low pressure system is located to the northwest of Scotland. The system moves towards east and deepens, resulting in an acceleration of the winds. At 06UTC (b) the strongest winds, exceeding 25 *ms*^−1^, struck the North Sea and the power production terminates (blue areas in the lower row in Fig. 5). As the center of the strong extratropical cyclone approaches Norway the mean wind speed increases further and reaches 35–40 *ms*^−1^.

**Fig. 6:**
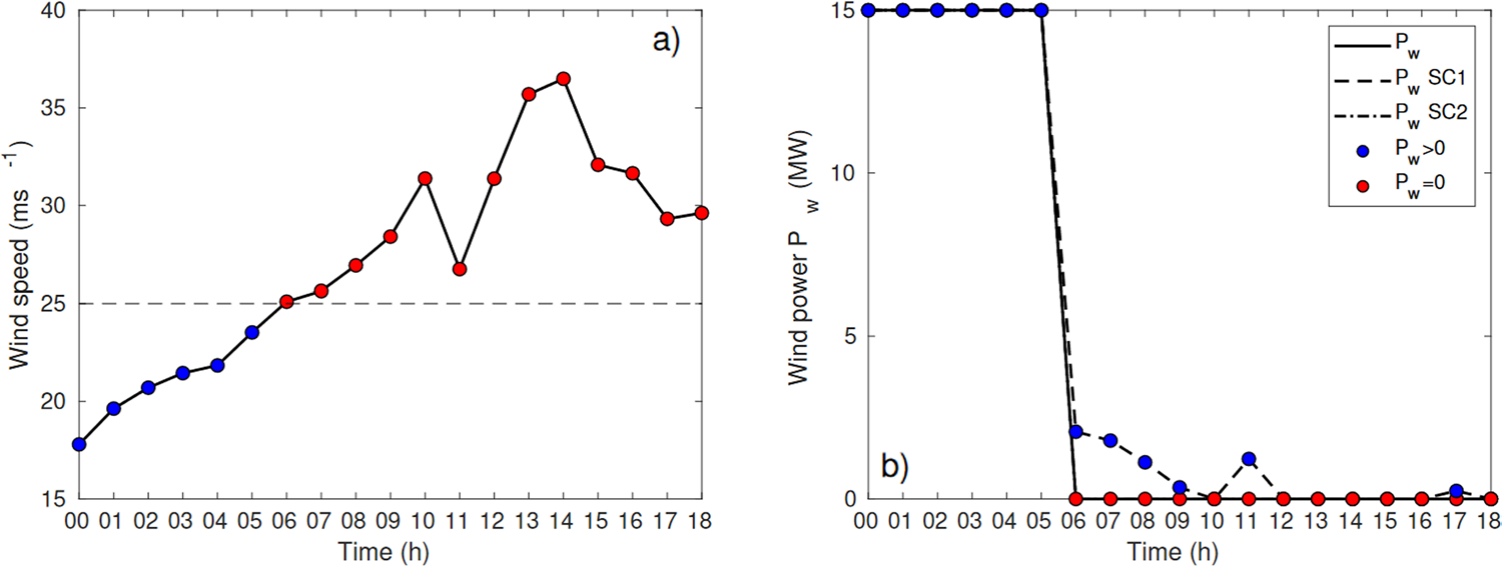
Time series of the wind speed (**a**) and wind power (*P*_*w*_) (**b**) for Sørlige Nordsjøen 2, between the 5th of December 2013 at 00UTC and 18UTC. Blue color indicate wind power production, while red color means terminated power production caused by too strong winds (u ≥ 25*ms*^−1^). *P*_*w*_ SC1 and *P*_*w*_ SC2 corresponds to power production using storm control 1 and 2, respectively.

The time series of the wind speed (a) and wind power (b) at a specific point in the offshore area “Sørlige Nordsjøen 2” (SN2) for the 5th of December from 00UTC to 18UTC are illustrated in Fig. 6. After 06UTC the wind is too strong for the power production to continue and the power production is terminated. This incident illustrates the wind power vulnerability towards strong winds. The figure also demonstrate the advantage of implementing storm control 1 (SC1, dashed line in Fig. 6) to exploit a larger fraction of the high wind speeds for power production.

**Table 4:** Short description of the MatLab functions used to calculate the variables in NORA3-WP.

Name	Type	Short description
WIND_WndDirSector.m	function	Calculates the mean of the prevailing wind-direction sector
WIND_calc_WndShear.m	function	Calculates the vertical wind shear
WIND_calc_WeibullParams.m	function	Calculates the Weibull shape, scale and standard deviation
WIND_calc_AbsRampRate.m	function	Calculates the absolute wind speed and wind power ramp-rates
WIND_calc_WndPowerDensity.m	function	Calculates the energy density in the air
WIND_calc_PowerDeliver.m	function	Calculates the wind power delivered by the air stream to the turbine rotor
WIND_calc_PowerCaptureCoeff.m	function	The efficiency of a turbine to extract the energy content in the air stream
WIND_calc_TurbinePowerProd.m	function	Calculate wind power production for a selected turbine and method
WIND_calc_FullLoadHours.m	function	Calculates the full load hours for a selected turbine
WIND_calc_CapacityFactor.m	function	Calculates the wind power capacity factor for a selected turbine
WIND_calc_PercCubedProd.m	function	Calculates the time fraction the power production is a function of the wind speed cubed
WIND_calc_PercRatedProd.m	function	Calculates the time fraction the power production is rated
WIND_calc_PercZeroProd.m	function	Calculates the time fraction the power production is zero

## Data Availability

NORA3-WP is created using MatLab (version 2018a). A short description of the functions used to create the variables in NORA3-WP can be found in Table 4. The matlab scripts are permanently archived at zenodo: 10.5281/zenodo.6138696.
